# Efficacy of telerehabilitation after spinal neurosurgery: a systematic review and meta-analysis

**DOI:** 10.1007/s11845-026-04403-1

**Published:** 2026-04-20

**Authors:** Tahir Keskin, İsmet Tümtürk, Fatih Özden, Emine İrem Şahin, Zeliha Başkurt, Ferdi Başkurt

**Affiliations:** 1 Faculty of Health Sciences, Department of Physiotherapy and Rehabilitation, Antalya Bilim University, Döşemealtı, Antalya, 07190 Turkey; 2 Köyceğiz Vocational School of Health Services, Department of Health Care Services, Muğla Sıtkı Koçman University, Muğla, Turkey; 3 Institute of Health Sciences, Department of Physiotherapy and Rehabilitation, Süleyman Demirel University, Isparta, Turkey; 4 Faculty of Health Sciences, Department of Physiotherapy and Rehabilitation, Süleyman Demirel University, Isparta, Turkey

**Keywords:** Telerehabilitation, Surgery, Spine, Review

## Abstract

**Background:**

The literature reports the postoperative effects of telerehabilitation following spinal surgery; however, no up-to-date systematic review and meta-analysis is available.

**Aim:**

The objective of this study was to present a systematic review and meta-analysis of telerehabilitation interventions after spinal neurosurgery.

**Methods:**

A literature search was conducted in electronic databases “ScienceDirect, PubMed and Web of Science (WoS)”. A total of 3806 studies were accessed using the identified keywords. According to the inclusion and exclusion criteria, a total of 7 articles were included. The “PEDro scale” was used to assess the methodological quality of the included studies. The study protocol has been registered in PROSPERO (Registration number: CRD42024586517).

**Results:**

Seven studies were included in the systematic review. The included studies were of “fair” quality based on the mean PEDro score. Pain and functional capacity were among the most assessed physical outcomes. Telerehabilitation showed low to moderate effectiveness on the Visual Analog Scale (VAS) and Numeric Pain Rating Scale (NPRS)-based pain severity (ES: -0.31, 95% CI: -6.15; 5.53). Besides, there is moderate to strong evidence for Oswestry Disability Index (ODI)-based disability scores on the effectiveness of telerehabilitation after lumbar spine surgery (LSS) (ES: -0.73, 95% CI: -12.01; 10.55).

**Conclusions:**

Telerehabilitation methods are effective, acceptable, and feasible in individuals after spinal neurosurgery. Telerehabilitation has been found to improve pain and disability in individuals after spinal neurosurgery.

**Supplementary Information:**

The online version contains supplementary material available at https://doi.org/10.1007/s11845-026-04403-1.

## Introduction

Spinal surgeries represent a crucial treatment avenue for patients who fail to respond to conservative interventions. In light of technological advancements, surgical procedures’ precision, safety, and efficacy have markedly improved [1]. Nevertheless, despite these advances, postoperative complications such as muscle dysfunction may occur due to the inherent nature of spinal neurosurgery [2]. Therefore, it is evident that postoperative rehabilitation plays a crucial role in the patient’s functional recovery.

Studies have demonstrated that rehabilitation in the postoperative period accelerates functional recovery, contributes to pain reduction, and provides more effective results than those observed in patients who do not receive postoperative rehabilitation [3, 4]. Furthermore, the efficacy of postoperative rehabilitation programs is contingent upon patient participation and completion. Despite the established advantages of rehabilitation, some patients are unable to complete these programs due to various factors, including difficulties in accessing rehabilitation services, the capacity of health institutions, costs, and the formation of waiting lists due to high demand [2]. In instances where rehabilitation is required but cannot be adequately provided, innovative solutions have emerged owing to technological advancements, facilitating more convenient access to rehabilitation services for patients [2, 5]. Telerehabilitation represents a pivotal strategy in this regard.

Telerehabilitation, a branch of telehealth, has been developed for the remote control and monitoring of rehabilitation using communication technologies. The potential benefits of telerehabilitation include ensuring the continuity of rehabilitation, delivering health services to geographically remote areas, saving time and resources, and its use in the treatment of many chronic diseases [6]. Telerehabilitation methods, which are also employed following spinal surgery with a prolonged recovery period, assist patients in managing their perioperative period more effectively. These interventions facilitate healthy lifestyle modifications, enhance medication adherence, abbreviate the recovery period in the postoperative phase through patient monitoring or protocol compliance, and augment patients’ self-care abilities, self-assurance, and satisfaction [2, 7–9]. Furthermore, the education provided to patients through digital health applications is more advantageous than written methods, as it incorporates visual and audio elements. This practical service allows patients to gain a deeper comprehension of rehabilitation protocols and practice independently in the clinic or at home [8].

The utilization of telerehabilitation following spinal neurosurgery has demonstrated favorable outcomes concerning patient education, rehabilitation participation, and feasibility, particularly within the postoperative period. Consequently, this study aimed to present a systematic review and meta-analysis of telerehabilitation interventions after spinal neurosurgery.

## Materials and methods

### Search strategy

The literature search was conducted in the “ScienceDirect, PubMed, and Web of Science (WoS)” databases in August 2024. The keywords used in the literature review were discussed among the authors, and a consensus was reached regarding the final version of the keywords used in the review. This systematic review and meta-analysis is based on the “Preferred Reporting Items for Systematic Reviews and Meta-Analyses (PRISMA)” [10]. The study protocol has been registered in PROSPERO (Registration number: CRD42024586517). The search was performed with a combination of “Boolean operators” (AND, OR). Title, abstract and keyword filtering were used in all databases during the search. The detailed search strategy is described in Supplementary Material 1 (see Supplementary Material 1).

### Eligibility criteria

The inclusion criteria were (a) full-text studies written in English and (b) studies involving postoperative telerehabilitation interventions given to patients undergoing neurosurgery in the spine region. Exclusion criteria were (a) studies including participants who underwent only surgical procedures without telerehabilitation intervention, (b) the presence of neurosurgical intervention due to trauma, malignancy, or deformity.

### Study selection and data extraction

**Fig. 1:**
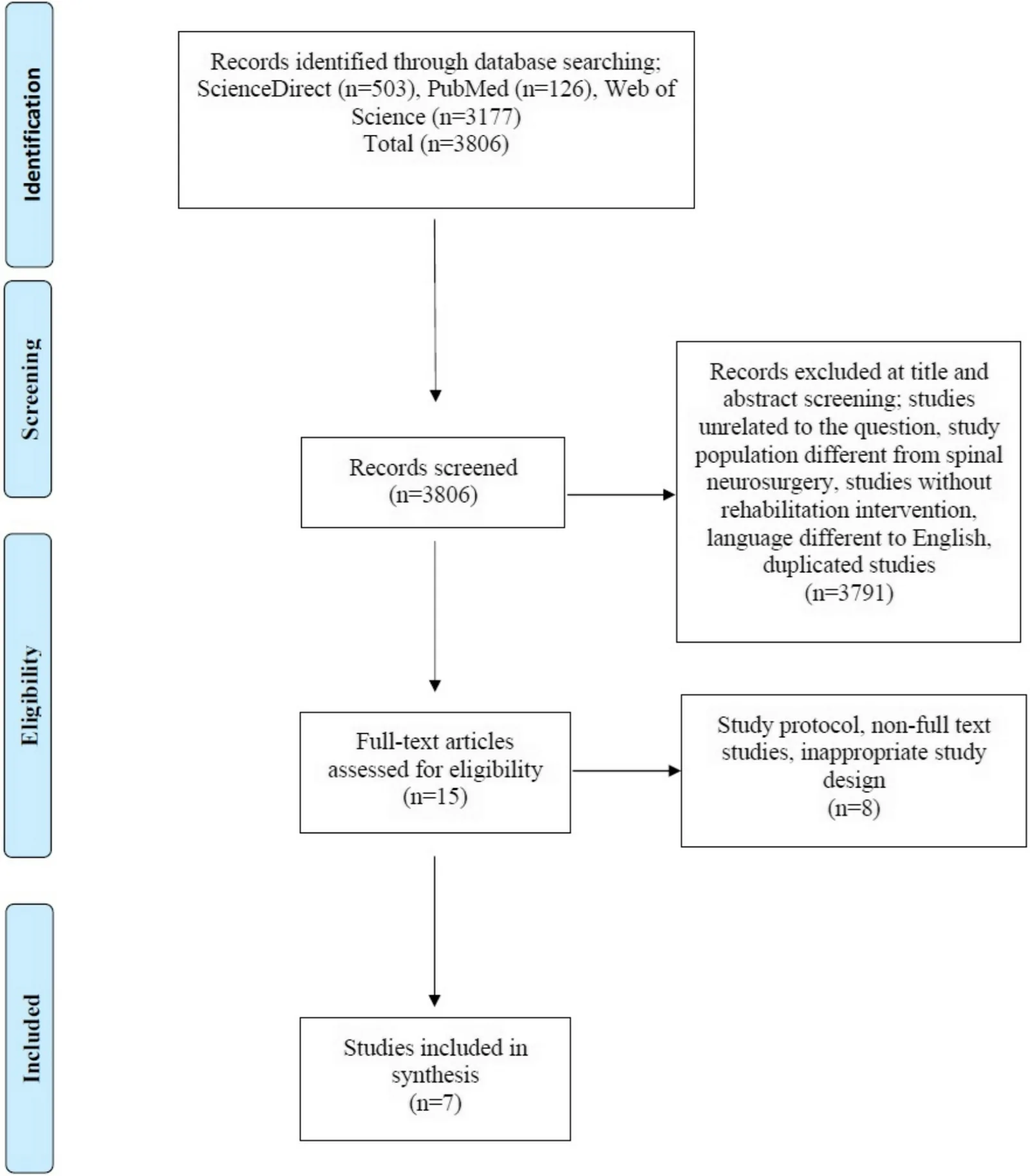
PRISMA flow diagram

All studies accessed with the identified keywords were exported to the software (Rayyan, Rayyan Systems Inc., USA). Rayyan is a practical software that provides an interface for selecting articles that meet the eligibility criteria in reviews. Rayyan’s responsiveness was reported by Ouzzani et al. [11]. A total of 3806 studies (ScienceDirect (*n* = 503), PubMed (*n* = 126), Web of Science (*n* = 3177)) were accessed by the two authors. Duplicate publications were detected by the Rayyan software and removed by the authors. Two researchers then independently screened the articles by reading the titles and abstracts according to the eligibility criteria. Study selection was determined by marking “exclude, include or maybe”. If there were any conflicting study selections between the two researchers, the final decision was finalized in consultation with the third researcher. Detailed study selection stages and numerical data are shown in Fig. 1. The following data were extracted from the articles included in the present review: Date of publication, type of study, diagnosis of participants, type of surgery, the objective of the study, number of participants, age of participants, gender of participants, interventions, outcome measures, follow-up and results (Table 1).

**Table 1:** Characteristics of included studies

Author year/ Design	Disease/Surgery	Objective	Sample characteristics	Interventions	Outcomes	Results
Debono et al., 2021 [7] Retrospective analysis of prospectively gathered data	Radiculopathy with disc prolapse ACDF with transverse cervical incision	To demonstrate a reduction in the LOS in hospital for ACDF without increasing the risk for patients by comparing 2 groups before and after ERAS implementation using propensity score matched analysis	IG ▪n:271 ▪Age: 47.3 ± 9.5 y ▪Male: 53% ▪CG ▪n:268 ▪Age:49.5 ± 10.7y ▪Male: 48%	IG: ERAS implementation: Pre- and postoperative education (including physio-education, early mobility and physiotherapy), follow-up and consultation with mobile application and telephone CG: Conventional group	▪LOS ▪Postoperative complications (minor, major) ▪Readmission and reoperation (day 30 and 90) ▪Satisfaction survey	▪In terms of LOS, IG (1.40 ± 0.6 d) had significantly shorter duration than CG (2.96 ± 1.35 d) (*p* < 0.001). ▪There was NSD between groups in terms of overall complications (*p* = 0.52), readmission (*p* = 1.000), reoperation (*p* = 0.49) and satisfaction survey (*p* > 0.05).
Erdogan & Bulut, 2020 [9] Randomized controlled study with a pre-test‒post-test design	LDH surgery	To identify the effects of computer assisted training schemes provided to patients undergone surgery for LDH on their level of knowledge, level of anxiety, problems experienced after discharge, and level of functional incapacity	IG ▪n:31 ▪Age: 39.97 ± 10.89 y ▪Male: 54.8% CG ▪n:31 ▪Age: 40.58 ± 11.45 y ▪Male: 58.1%	IG: Computer assisted training and counselling via training website CG: Training guidebook with the same concept of the website	▪Information level ▪ODI (functional capacity) ▪STAI (anxiety) ▪Problems following discharge (15th day, 1st and 3rd months after surgery)	▪Information level was significantly increased in both groups after admission (*p* = 0.001) and information level was in favor of IG for all of time (*p* = 0.001, *p* = 0.003). ▪In terms of ODI, both groups showed significant increase in functional capacity (*p* = 0.001). The increase in IG was higher than CG (15th day, *p* = 0.023; 1st month, *p* = 0.013; 3rd month, *p* = 0.009). ▪According to STAI, anxiety scores significantly decreased over time for both groups (*p* = 0.001), except for trait anxiety scores of CG (*p* = 0.225). Anxiety scores of the IG was lower than CG over time (*p* = 0.001, *p* = 0.002). ▪Pain, pain in the legs, sleep disorders, loss of sensation, decrease in social activities and difficulties in maintaining a normal routine in working life were the same frequent problems after discharge.
Hou et al., 2019 [2] Multicenter, prospective randomized controlled trial	Patients with LDH, spinal stenosis, lumbar spondylolisthesis Lumbar spinal surgery	To examine the efficacy of mobile phone-based rehabilitation systems in patients who underwent lumbar spinal surgery	IG ▪n:84 ▪Age: 51.11 ± 9.54y ▪Male: 43% CG ▪n:84 ▪Age: 49.36 ± 9.52y ▪Male: 50%	IG: Mobile phone-based eHealth program (including videos, reminder, feedback, communication) ▪Began 3 months after surgery ▪Twice a day*20 m ▪At least 2 months CG: Usual care (including physical advice and to train the back muscles)	▪ODI (function) ▪VAS (pain) ▪Likert scales, EuroQoL-5D, SF-36 (General mental health and Quality of life) (3, 6, 12, 24 months)	▪In terms of ODI and VAS, IG had significantly higher improvement in function and pain level than CG at 24 months after surgery (*p* = 0.03), but there was NSD between groups at other time points (*p* > 0.05). According to subgroup analysis, improvements in ODI and VAS were found more significant in those completed 6 or more sessions each week than CG at 6th, 12th and 24th months (*p* < 0.05). ▪There was SSD as for improvement in Likert scale for movement at 24 months (*p* = 0.01), EuroQoL-5D scores at 6th (*p* < 0.001), 12th (*p* = 0.003) and 24th months (*p* = 0.001), SF-36 general health and physical functioning scores except for 12th month (*p* = 0.02, *p* = 0.004) in favor of IG compared CG.
Murray et al., 2022 [16] Single-arm pilot study	Adolescents with idiopathic spinal deformities and their parents Major spinal fusion surgery	To evaluate the feasibility and acceptability of the first Internet-based psychological intervention delivered during the perioperative period	▪n:26 (13 adolescent–parent dyads) ▪Age of adolescents: 14.3 ± 1.4 y ▪Male: 30.8%	IG: Online Internet-delivered psychosocial intervention including six lessons (20 to 30 m) delivering during the month prior to and the month following surgery ▪Preoperative phase began 4 to 6 w before surgery ▪Postoperative phase began 1 w after surgery	▪ Feasibility (recruitment rate, intervention engagement, measure completion (*PROMIS Pain Intensity and Interference scales *PedsQL-Short Form *STAI *Adolescent Sleep Wake Scale *Pain Coping Questionnaire *Brief Symptom Inventory) ▪Acceptability (quantitative ratings and qualitative interviews) (4–8 w before surgery; 1 w before surgery; 6–8 w post-surgery; 3 months post-surgery)	▪The overall participation rate was 68.4%, and the participation rate among those approached was 81.2%. Dyads completed all 3 preoperative and postoperative sessions, and engagement in the online program was found to be excellent. ▪During the intervention, 91.7% of adolescents (*n* = 11/12) and 83.3% of parents (*n* = 10/12) completed at least five out of six telephone coaching calls. ▪Assessment completion was excellent. All dyads completed T1 and T2 assessments. Of the remaining 12 dyads, 100% completed the T3 and T4 assessments. ▪Overall, moderate to high levels of quantitative acceptability were reported for the preoperative and postoperative program. The qualitative feedback from participants about their intervention experiences was reported according to the purposes of (1) intervention components, (2) general program structure, and (3) suggestions for improvement.
Sadeghpour Ezbarami et al., 2023 [15] Randomized controlled trial	Patients with LDH surgery	To evaluate the impact of a mobile-based educational program on patients’ postoperative care for LDH surgery	IG ▪n:75 ▪Age: 43.34 ± 9.25y ▪Male: 47.1% CG ▪n:75 ▪Age: 42.51 ± 8.30y ▪Male: 62%	IG: Learning with LUCAT (information about LDH, postoperative care, daily activities, wound management, sexuality, videos, photos, messages, consultations, etc.) ▪8 w CG: No educational content of LUCAT	▪ Validated questionnaire (participants’ knowledge, attitudes, and practices of postoperative care for LDH surgery) (Baseline, immediately after program completion, 8w after program completion)	▪As for the effect of the intervention on the variables, the results showed that the effect of group (*p* < 0.001), the effect of time (*p* < 0.001), and the interaction (*p* < 0.001) was significant. ▪After intervention and in the 8-w following the study, knowledge, attitudes, and practices scores in the IG were significantly higher than CG (*p* < 0.001).
Saha & Goktas, 2021 [8] Randomized controlled experimental study	Patients underwent lumbar disc surgery	To determine the effect of computer-based discharge training on patients with lumbar disc surgery on self-care agency and independence in DLA	IG ▪n:30 ▪Age: 48.93 ± 8.50y ▪Male: 50% CG ▪n:30 ▪Age: 49.03 ± 9.93y ▪Male: 63.3%	IG: Computer-based discharge training (safety, breathing-coughing and other exercises, correct body mechanics in DLA, returning to work, etc. and a CD content ▪6w CG: Routine discharge in ward (lying, transfers, safe environment)	▪ MBI (functional status) ▪ ESCA (self-care ability)	▪The increase in the MBI and ESCA scores after training in both groups were statistically significant (*p* < 0.001). ▪The increase (*p* = 0.001) and mean scores (*p* = 0.036) of ESCA was found to be higher in the IG than CG. ▪There was no difference between the MBI mean scores before and after the training between the groups (*p* > 0.05).
van der Horst et al., 2024 [5] Non-randomized experimental designed, qualitative, and quantitative pilot study	Patients undergoing decompression or spinal fusion surgery	▪To explore the potential benefits for patients undergoing spinal surgery of the digital ACT and PP intervention Strength Back ▪To explore the feasibility of a future randomized controlled trial in terms of recruitment and dropout ▪To assess the acceptability of Strength Back by patients undergoing spinal surgery	IG ▪n:17 ▪Age: 62 ± 12y ▪Male: 65% CG ▪n:20 ▪Age: 66 ± 14y ▪Male: 40%	IG: A digital health intervention (Strength Back) based on ACT and PP + Usual care CG: Usual care (brochures containing information, physical guidelines, etc.; and appointments: before surgery, 10 days after surgery by phone, physical checkup 6w (both surgery types) and 12 w (spinal fusion surgery) after surgery	▪NPRS (pain intensity) ▪MPI (pain interference) ▪HADS (depression and anxiety) ▪ELS (valued living) ▪PIPS (psychological flexibility) ▪MHC-SF (mental wellbeing-emotional, social, psychological, total) ▪Recruitment, dropout rates, dropout characteristics (feasibility)▪Semi structured interviews combined with log data (acceptability) ▪ TWEETS (acceptability)	▪Except for ELS, both groups showed significant improvement in the NPRS (week average and worst), HADS, MPI, and PIPS total score (p: <0.001, *p* < 0.02). In the IG, the total (*p* = 0.004) and emotional well-being (*p* = 0.03) increased significantly over time; but there was no significant difference between MHC-SF total and sub scores the pre- and posttest times in the CG. ▪Between-group comparisons, the IG showed a significantly larger decline in NPRS score than CG (*p* = 0.003). ▪51% (18/35) of the approached patients did not want to participate and did not start with or dropped out. Of these 18 patients, 14 (78%) were female and 14 (78%) underwent decompression surgery. ▪Weekly modules on average 80% (12/15) was completed by patients undergoing spinal fusion and 67% (6/9) was completed by patients undergoing decompression surgery. Most participants (15/17, 88%) in the IG would recommend the intervention to future patients. ▪The mean total score of all participants in the IG on the TWEETS was 2.6 out of a possible 4 (65% of the maximum score). ▪A total of 15 weekly modules were offered to the patients undergoing spinal fusion (6/17, 35%). Log data of these patients showed that the average number of weekly modules completed was 10.2 out of 15.

*ACDF* anterior cervical discectomy and fusion, *ACT* acceptance and commitment therapy, *CG* control group, *DLA* daily living activities, *ELS* Engaged Living Scale, *ERAS* enhanced recovery after surgery, *ESCA* Exercise of Self-Care Agency Scale, *EuroQoL-5D* Euro Quality of Life – 5 Dimension Health Questionnaire, *HADS* Hospital Anxiety and Depression Scale, *IG* intervention group, *LDH* lumbar disc herniation, *LOS* length of stay, *LUCAT* LUmbar CAring Training-app, *MBI* Modified Barthel Index, *MHC-SF* Mental Health Continuum–Short Form, *MPI* Multidimensional Pain Inventory, *n* number, *NPRS* Numeric Pain Rating Scale, *NSD* not significant difference, *ODI* Oswestry Disability Index, *PedsQL* Pediatric Quality of Life Inventory, *PIPS* Psychological Inflexibility in Pain Scale, *PP* positive psychology, *SF-36* Short Form- 36 Health Survey, *SSD* statistically significant difference, *STAI* State-Trait Anxiety Inventory, *TWEETS* Twente Engagement With eHealth Technologies Scale, *VAS* Visual Analog Scale, *w* weeks, *y* years, *d* days

### Quality assessment

Quality assessment of the included studies was based on the PEDro scale. The PEDro scale consists of 11 questions measuring the quality of studies (e.g. randomization, blinding). All items of the PEDro scale were rated as “Yes” or “No”. Each “Yes” answer is scored 1 point. But even if the first question is answered “Yes”, it is not included in the total score. According to the PEDro total scores, 9–10 was defined as “excellent”, 6–8 as “good”, 4–5 as “fair” and 0–3 as “poor” [12].

### Meta‑analysis

The meta-analysis of the review was conducted using the “Meta-Mar web application (Philipps-Universität Marburg, Germany)” [13]. The “Standardized Mean Difference (SMD)” was calculated with “the mean, standard deviation (SD), and sample sizes”. The “Cochrane Handbook” was used to identify “unknown SD (change over time) and confidence interval (CI) of delta values, reference values” [14]. The meta-analysis results were provided with “SMD, CI, weighted average effect size, and p-value” scores. In addition, “I2, Tau2, and Chi2” values ​​were also given.

## Results

### Screening results

After the deletion of duplications, a total of 1642 articles were screened. Finally, seven studies were included in the systematic review and the meta-analysis.

### Study characteristics

The characteristics of the studies in this systematic review and meta-analysis are shown in Table 1. All the studies were published in English between 2019 and 2024. Four studies were designed as randomized controlled trials [2, 8, 9, 15], two were designed as pilot feasibility and acceptability studies (5, 16), and one of them was a retrospective analysis [7]. The included studies were aimed to investigate the feasibility and acceptability of mobile phone or computer-based telerehabilitation interventions in patients with undergone spine surgeries (such as spinal fusion, decompression, or discectomy surgeries), to evaluate the effects and to compare with controls (e.g., conventional or usual care or routine discharge training including brochures, guidebooks and advices).

### Methodological quality assessment

According to the PEDro scoring of the seven studies reviewed with final evaluations (Table 2), PEDro scores ranged from 3 to 8 (median = 6; mean = 5.42 ± 1.9); and four articles were “good” [2, 8, 9, 15], 1 article was “fair” [5], two articles were ‘‘poor’’ [7, 16] quality. Based on the mean score of the studies, a ‘‘fair’’ level of quality was considered in general according to the PEDro classification [17]. Based on whether the PEDro scale items were provided in the included studies, while “eligibility criteria” and “point estimates and variability’’ items were found in all of these studies ‘‘blind subjects’’ and ‘‘blind therapists’’ were mentioned none. Two studies had ‘‘concealed allocation’’ [2, 15], and only one had ‘‘blind assessors’’ [2]. In 3 studies, there was no ‘‘random allocation’’ method [5, 7, 16]. One study did not use the ‘‘baseline comparability’’ [16]; one did not have ‘‘adequate follow-up’’ and ‘‘intention to treat analysis’’ [7]. Lastly, one of the studies did not have ‘‘between group comparisons’’ [16].

**Table 2:** PEDro scores

Study	Q1	Q2	Q3	Q4	Q5	Q6	Q7	Q8	Q9	Q10	Q11	Total
Debono et al., 2021	Y	N	N	Y	N	N	N	N	N	Y	Y	3
Erdogan & Bulut, 2020	Y	Y	N	Y	N	N	N	Y	Y	Y	Y	6
Hou et al., 2019	Y	Y	Y	Y	N	N	Y	Y	Y	Y	Y	8
Murray et al., 2022	Y	N	N	N	N	N	N	Y	Y	N	Y	3
Sadeghpour Ezbarami et al., 2023	Y	Y	Y	Y	N	N	N	Y	Y	Y	Y	7
Saha & Goktas, 2021	Y	Y	N	Y	N	N	N	Y	Y	Y	Y	6
van der Horst et al., 2024	Y	N	N	Y	N	N	N	Y	Y	Y	Y	5
Total	7	4	2	6	0	0	1	6	6	6	7	

**Q1**: Eligibility criteria; **Q2**: Random allocation; **Q3**: Concealed allocation; **Q4**: Baseline comparability; **Q5**: Blind subjects; **Q6**: Blind therapists; **Q7**: Blind assessors; **Q8**: Adequate follow-up; **Q9**: Intention-to-treat analysis; **Q10**: Between-group comparisons; **Q11**: Point estimates and variability

### Outcome measures

Outcome measures are presented in Table 1. Functional capacity was evaluated with the Oswestry Disability Index (ODI) [2, 9] and the Modified Barthel Index (MBI) [8] in three studies. Besides functional capacity, self-care ability was evaluated with Exercise of Self-Care Agency Scale (ESCA) in one of them [8]. Pain was one of the most evaluated physical outcomes. Visual Analog Scale (VAS) and Numeric Pain Rating Scale (NPRS) were used to evaluate pain severity, and Multidimensional Pain Inventory (MPI) was used to evaluate pain interference [2, 5]. In addition to the physical outcomes, various psychological outcomes were evaluated. Depression and anxiety levels were assessed with Hospital Anxiety and Depression Scale (HADS) and State-Trait Anxiety Inventory (STAI) [5, 9]. General mental health, quality of life (Likert Scale, Euro Quality of Life – 5 Dimension Health Questionnaire (EuroQoL-5D), Short Form-36 (SF-36) [2], valued living (Engaged Living Scale (ELS), psychologycal flexibility (Psychological Inflexibility in Pain Scale (PIPS) and mental wellbeing (Mental Health Continuum–Short Form (MHC-SF)) [5] were assessed in other studies.

Two of the included studies evaluated patients’ knowledge/information levels about situation [9, 15], one evaluated patients’ attitudes and practices of postoperative care [15]. Two studies examined postoperative complications and problems following discharge [7, 9]. Length of stay in hospital, readmission and reoperation in first 90 days, and satisfaction level were recorded also in one of the studies [7].

In addition, feasibility and acceptability were assessed in two of the studies [5, 16]. Recruitment and dropout rates, dropout characteristics, intervention engagement and measure completion were used to assess the feasibility. Acceptability was assessed with interviews and Twente Engagement with eHealth Technologies Scale (TWEETS) [5, 16].

### Effectiveness of telerehabilitation on cervical spine surgeries

Only one of the included studies used telerehabilitation after cervical spine surgery and reported that intervention reduced hospital stays more than conventional treatment without any additional complication or increased treatment satisfaction [7].

### Effectiveness of telerehabilitation on lumbar spine surgeries

As for lumbar spine surgeries, three studies showed that mobile or computer based telerehabilitation provided significantly higher increase in functional capacity evaluated with ODI and MBI; and self-care ability; and general physical health and quality of life evaluated with Likert Scale, EuroQoL-5D and SF-36 than standard training methods including verbal education and guidebooks [2, 8, 9]. In addition, high information level was correlated with lower ODI scores at 1st month in telerehabilitation group and information level was significantly higher than training guidebook group over the time [9]. In line with this, the other study reported that a significantly higher knowledge, attitude and practice levels than controls following 8 weeks smartphone-based education program after lumbar surgery [15]. Additionally, individuals had lower anxiety levels according to STAI after telerehabilitation, and according to the results of the study, a significant correlation was observed between ODI and state anxiety scores; information level and anxiety level were negatively correlated in both groups [9]. After intervention including core stability exercise applied via mobile phone based system, ODI and VAS-pain scores showed higher improvement at 24th months after surgery than usual care. Also, these improvements were more significant in those with high compliance at 6th, 12th and 24th months [2]. In another study, it was reported that pain improved significantly more according to the NPRS score after telerehabilitation compared to usual care [5]. Moreover, while there was no significant improvement in mental well-being after usual care, significant improvements were observed after telerehabilitation [5].

### Feasibility and acceptability of telerehabilitation after spine surgery

One study showed the feasibility and acceptability of internet-based psychological intervention including cognitive-behavioral therapy skills targeting anxiety, sleep, and acute pain management in adolescents undergone major spinal fusion surgery and their parents. Participation rate (81.2%), intervention engagement for adolescents and parents (91.7% and 83.3%, respectively) and measure completion (100%) criteria were met for feasibility and moderate to high levels of acceptability was observed [16]. Another study mentioned the feasibility and acceptability of a digital ‘‘acceptance and commitment therapy’’ and ‘‘positive psychology’’ based health intervention after decompression or spinal fusion surgery. As for the feasibility of a future RCT, 51% (18/35) of the approached patients did not want to participate and did not start with or dropped out of the intervention. Of these 18 patients, 14 (78%) were female and 14 (78%) underwent decompression surgery. Female sex, decompression surgery and > 65 years were the frequent dropouts and/or nonadherences characteristics [5]. As for the acceptability, of the available weekly modules on average 80% was completed by patients undergoing spinal fusion and 67% was completed by patients undergoing decompression surgery. A total of 68% of the participants used the intervention until the final interview. Some patients found intervention more accessible and more reliable than brochure. In terms of TWEETS score the intervention were considered easy to use, helpful and fitting. Most participants (88%) would recommend the intervention to future patients [5]. As to content of the intervention, the tips from previous patients and pain medication module were appreciated by 65% of the participants. Hospital contact details were found clear and useful. However, physical therapy guidelines were not satisfied by 18% of the participants. Psychological content was too high and more specific physical guidelines, more physical therapy exercises, and information about returning to work were demanded [5].

### Meta-analysis results

Our meta-analysis of seven studies found that telerehabilitation had a low impact on pain severity and disability scores. Two studies followed up on pain scores at three months. In addition, disability scores were considered during the post-op three months. The I2 values of the pain (89%) and disability (95%) score pooling were lower than 0.50%. Therefore, we focused on the random effect models. Specifically, telerehabilitation showed low to moderate effectiveness on VAS/NPRS-based pain severity (ES: -0.31, 95% CI: -6.15; 5.53). Besides, there is moderate to strong evidence for ODI-based disability scores on the effectiveness of telerehabilitation after lumbar spine surgery (LSS) (ES: -0.73, 95% CI: -12.01; 10.55). These pooled findings are summarized in Table 3.

**Table 3:**
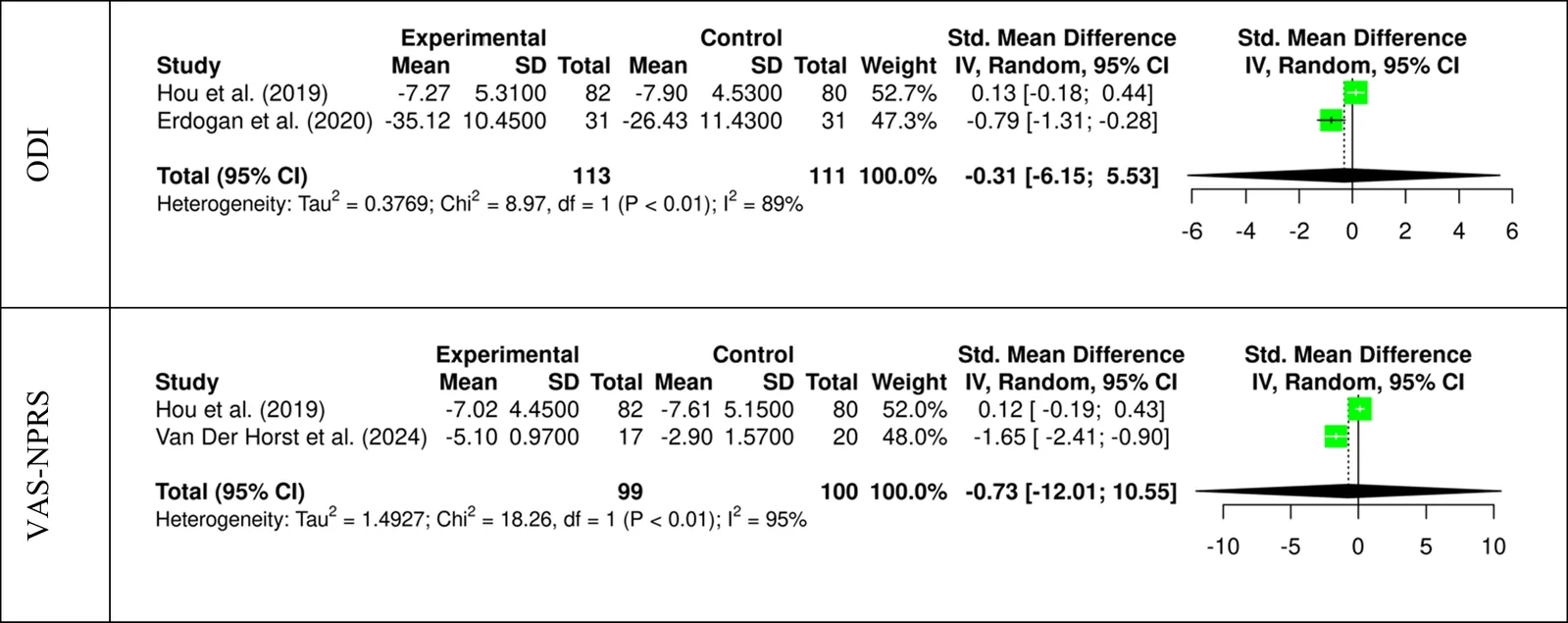
Forest plots of the ODI and VAS/NPRS score at three months

### Adverse events

Only one study reported adverse effects in both telerehabilitation and control groups, including mild joint and back pain. However, these complaints did not differ in frequency and severity between groups (*p* > 0.05) [2].

## Discussion

The present systematic review and meta-analysis aimed to report the level of effectiveness of telerehabilitation interventions after spinal neurosurgical surgery and to provide meta-analysis results on common parameters. Adherence to and engagement in rehabilitation strategies is required to optimize recovery after spinal neurosurgical surgeries. This can be achieved by telerehabilitation. In addition, telerehabilitation strategies are preferred after spinal neurosurgical surgeries for many reasons such as reducing the risk of nosocomial infection, facilitating access to rehabilitation, and cost-effectiveness. To the best of our knowledge, a systematic review of telerehabilitation studies after spinal neurosurgery has been conducted previously, but meta-analysis is not available in the literature and there is a need for a recent review study on this issue. An up-to-date review and meta-analysis of telerehabilitation methods and outcomes after spinal neurosurgery can contribute to the literature. This systematic review and meta-analysis provide evidence for the effectiveness of telerehabilitation therapy after spinal neurosurgery. The included studies were designed as a randomized controlled trial, pilot feasibility and acceptability study and retrospective analysis. The findings from our present systematic review and meta-analysis indicate moderate quality on the PEDro scale, feasibility and acceptability of telerehabilitation methods, pain and functional capacity were among the most commonly used outcome measures, telerehabilitation reduced the length of hospital stay, telerehabilitation was more effective than verbal advice, and brochures in terms of function and quality of life, high levels of knowledge and low levels of anxiety provided by telerehabilitation are associated with a reduction in disability, the improvement in pain and disability provided by telerehabilitation is maintained in the long term, with low to moderate evidence for pain and moderate to strong evidence for disability.

The majority of the reviewed studies had good scores on the PEDro scale [2,8,9,15]. However, only one of the studies was blinded [2]. This may increase the risk of bias. Future studies should use blinding methods to avoid potential bias and improve the level of evidence. In addition, four of the studies included in the review were randomized controlled trials [2,8,9,15]. We recommend focusing on more randomized controlled trials. Pain assessment was one of the most used outcome measures. Previous reviews have also addressed the effects of telerehabilitation on pain and found parallel results with the present review. A review of the effectiveness of telerehabilitation for shoulder pain reported a low level of evidence for pain and disability [18]. An overview of systematic reviews investigated the effects of telerehabilitation on musculoskeletal pain. Most of the reviewed reviews reported evidence that pain levels improved with telerehabilitation [19]. In another review, all interventions and measurement methods of telerehabilitation in pain management were analyzed in detail and the authors reported that telerehabilitation can be successfully employed in pain management [20]. According to the meta-analysis results of our study, we found that telerehabilitation after spinal neurosurgery is at a low to moderate level of evidence in terms of pain. The reason for this positive effect of telerehabilitation on pain may be that a wide range of rehabilitation options can be applied in a short time and with high compliance due to its easy accessibility and feasibility. Another reason may be that the post-surgical knowledge levels of individuals related to surgery are optimized through telerehabilitation and that it is a kind of pain school. Individuals who have undergone spinal neurosurgery may not be able to make frequent hospital visits after discharge, but telerehabilitation can provide patient education and rehabilitation at any time by using powerful expression methods.

Another outcome measure used in telerehabilitation studies after spinal neurosurgery is the level of disability. The disability results of the present review and similar previous studies are in parallel. A study reviewing telerehabilitation methods in chronic neck pain reported that telerehabilitation was superior in improving pain and disability. However, it was also reported that there was no significant difference between face-to-face intervention groups and telerehabilitation groups [21]. Based on this finding, we emphasize that telerehabilitation may be essential in terms of disability in individuals undergoing spinal neurosurgery, since it is assumed that a condition such as spinal neurosurgery surgery, which can be considered a more serious problem than chronic neck pain, cannot have access to continuous face-to-face intervention. In another study, the authors reported that telerehabilitation has positive effects on disability and can be used in individuals with musculoskeletal disorders [22]. In low back pain, telerehabilitation services with exercise videos have also been reported to improve disability [23]. In the results of our study, we also report moderate to high evidence for disability. In addition, high levels of knowledge and low levels of anxiety are associated with a reduction in disability. The positive effect of telerehabilitation on disability may be due to the optimal implementation of specific exercises given after spinal neurosurgery surgeries by the participants. Due to the nature of telerehabilitation, the fact that the participants had access to the necessary postoperative information at any time and that this information could be constantly updated by the clinician may have positively affected the level of disability. Therefore, we recommend that specific physical instructions, exercises, knowledge, attitudes and intervention strategies be implemented through telerehabilitation in this population.

Included studies of telerehabilitation after spinal neurosurgical surgeries also evaluated psychometric methods (depression, anxiety, quality of life, general mental health, knowledge of individuals about the condition). A previous review study reported that providing telerehabilitation to caregivers of stroke survivors improved caregiver burden, knowledge, competence and mood [24]. Another review reported that telerehabilitation improved quality of life in individuals with chronic diseases, but did not show an advantage over face-to-face interviews in terms of anxiety and depression [25]. The reason for this result may be that in individuals with chronic diseases such as diabetes and cancer, bad feelings such as long-term depression and anxiety may have settled. However, the population in our study showed improvements in psychometric outcomes with multiform telerehabilitation methods such as adequate information and correct exercise after neurosurgical surgery. We recommend multiform telerehabilitation methods to improve psychometric outcomes after spinal neurosurgery. The studies in the present review that measured the feasibility and acceptability of telerehabilitation after spinal neurosurgical surgery showed moderate to high levels of acceptability. In addition, the feasibility and acceptability of internet-based telerehabilitation methods are also demonstrated. However, female gender, decompression surgery and age > 65 years were common dropout and/or non-adherence factors for telerehabilitation. Participants found telerehabilitation interventions more accessible and reliable than the brochure. Finally, participants indicated that the psychological content of telerehabilitation interventions was too high and requested more specific physical instructions, more physical therapy exercises and information about returning to work. In a previous review assessing acceptance and satisfaction with telerehabilitation in stroke survivors, participants reported good acceptance, usability and satisfaction with telerehabilitation [26]. In the present review, we report results in line with previous results in terms of feasibility and acceptability. Although some barriers to the use of telerehabilitation have been reported [27], its effectiveness and simplicity have been recognized even in extreme situations such as pandemics. The applicability, acceptability and satisfaction level of telerehabilitation in the population undergoing spinal neurosurgery in the present review was good, as the mean age of the participants was not high. Considering the ever-improving technological literacy, we report that telerehabilitation methods are feasible in this population. In addition, telerehabilitation shortened the length of hospital stay after spinal neurosurgery. The content of telerehabilitation interventions in the reviewed studies; patient, parent, caregiver information, explanation of all stages of the surgical procedure, information about prescribed medications, information about the stages after discharge, continuous accessibility of clinicians, reminder notifications, video-assisted information approaches such as exercises, feedback to clinicians, muscle strengthening exercises, mobility exercises, core stability exercises, balance coordination exercises, functional activities, cognitive behavioral therapy, anxiety, sleep quality, coping with pain, thought stopping, relaxation strategies, activity pacing, interdisciplinary approaches, wound management, sexuality, messages, fall prevention training, ergonomics, nutrition recommendations, body mechanics principles, driving, return to work, positive psychology. A previous review focusing on telerehabilitation interventions in children with disabilities reported a great diversity in telemonitoring interventions [28]. We also report a wide diversity of telerehabilitation interventions after spinal neurosurgery. Especially interdisciplinary interventions can be effectively implemented with telerehabilitation. Approaches based on the biopsychosocial model can also be used through telerehabilitation. In addition, telerehabilitation interventions consider the ease with which clinicians and patients can communicate with each other and easily access treatment programs. The diversity of these interventions that can be implemented with telerehabilitation can be advantageous for clinicians, researchers and the current population. However, individuals after spinal neurosurgery wanted more emphasis on specific physical guidelines, physical therapy exercises and information about returning to work. All these advantages of telerehabilitation, such as diversity and accessibility, may have contributed to improved outcome measures for participants. Telerehabilitation methods are as diverse as telerehabilitation interventions. These include mobile applications, internet-based software, computer-generated software, phone calls, various notifications, communication networks, video visual tools, different storage devices and ensuring continuity of access to intervention protocols. Future studies may use different and more technological telerehabilitation methods such as instant assessments with sensors, exercise training and information with three-dimensional visualizations, and artificial intelligence-supported applications.

### Clinical implications

Telerehabilitation following spinal neurosurgery can be considered and implemented as a practical and effective alternative to standard rehabilitation. Telerehabilitation may improve access to rehabilitation, rehabilitation adherence, and long-term improvement, particularly for patients unable to attend face-to-face rehabilitation sessions. Telerehabilitation interventions that include structured exercise programs, such as patient education and a biopsychosocial approach, may offer greater improvements in functional recovery and quality of life. Additionally, the ease of continuous monitoring and feedback may help optimize postoperative outcomes. In addition to clinicians, healthcare systems and policymakers should consider integrating telerehabilitation into standard postoperative care to reduce the burden on healthcare services, shorten hospital stays, and improve overall rehabilitation efficiency.

### Limitations

Some limitations can be mentioned in our study. First, we could not create a homogeneous data pool due to the differences in the age and gender of the reviewed studies. Second, pooling of some parameters could not be performed due to heterogeneous data distribution. Third, the number of randomized controlled trials in the relevant field was limited. With the increase in the number of studies in the future, studies in which only randomized controlled trials are reviewed and meta-analyzed may yield more significant results.

## Conclusions

In conclusion, telerehabilitation methods are effective, acceptable, and feasible for individuals after spinal neurosurgery. Among individuals after spinal neurosurgery, telerehabilitation has low to moderate evidence for improving pain and moderate to strong evidence for improving disability. There is a wide range of interventions that can be implemented with telerehabilitation. Future studies should use various technological telerehabilitation methods in order to obtain more practical outcomes.

## Supplementary Information

Below is the link to the electronic supplementary material.

Supplementary Material 1 (DOCX 30.4 KB)

## Data Availability

The datasets used and analyzed during the current study are available from the corresponding author on reasonable request.
