# Supplementary material for: Efficacy of telerehabilitation after spinal neurosurgery: a systematic review and meta-analysis
Source: Ir J Med Sci. 2026 Apr 20;195(4):2271–83. doi: 10.1007/s11845-026-04403-1 (PMC13588874; doi:10.1007/s11845-026-04403-1)
Supplement: Supplementary file 1 [file 11845_2026_4403_MOESM1_ESM.docx]

**Supplementary Material 1 – Search strategies**

**Search strategy**

| **Search ID#** | **Search Terms** | **Search Options** |
| --- | --- | --- |
| S1 | cervical AND surgery AND tele | Boolean/Phrase |
| S2 | cervical AND surgery AND ehealth | Boolean/Phrase |
| S3 | cervical AND surgery AND mhealth | Boolean/Phrase |
| S4 | cervical AND surgery AND remote | Boolean/Phrase |
| S5 | cervical AND surgery AND internet | Boolean/Phrase |
| S6 | cervical AND surgery AND mobile | Boolean/Phrase |

428 references were included from ScienceDirect, PubMed and Web of Science

**Search strategy**

| **Search ID#** | **Search Terms** | **Search Options** |
| --- | --- | --- |
| S1 | cervical AND operation AND tele | Boolean/Phrase |
| S2 | cervical AND operation AND ehealth | Boolean/Phrase |
| S3 | cervical AND operation AND mhealth | Boolean/Phrase |
| S4 | cervical AND operation AND remote | Boolean/Phrase |
| S5 | cervical AND operation AND internet | Boolean/Phrase |
| S6 | cervical AND operation AND mobile | Boolean/Phrase |

65 references were included from ScienceDirect, PubMed and Web of Science

**Search strategy**

| **Search ID#** | **Search Terms** | **Search Options** |
| --- | --- | --- |
| S1 | cervical AND discectomy AND tele | Boolean/Phrase |
| S2 | cervical AND discectomy AND ehealth | Boolean/Phrase |
| S3 | cervical AND discectomy AND mhealth | Boolean/Phrase |
| S4 | cervical AND discectomy AND remote | Boolean/Phrase |
| S5 | cervical AND discectomy AND internet | Boolean/Phrase |
| S6 | cervical AND discectomy AND mobile | Boolean/Phrase |

49 references were included from ScienceDirect, PubMed and Web of Science

**Search strategy**

| **Search ID#** | **Search Terms** | **Search Options** |
| --- | --- | --- |
| S1 | cervical AND decompession AND tele | Boolean/Phrase |
| S2 | cervical AND decompession AND ehealth | Boolean/Phrase |
| S3 | cervical AND decompession AND mhealth | Boolean/Phrase |
| S4 | cervical AND decompession AND remote | Boolean/Phrase |
| S5 | cervical AND decompession AND internet | Boolean/Phrase |
| S6 | cervical AND decompession AND mobile | Boolean/Phrase |

72 references were included from ScienceDirect, PubMed and Web of Science

**Search strategy**

| **Search ID#** | **Search Terms** | **Search Options** |
| --- | --- | --- |
| S1 | cervical AND postoperative AND tele | Boolean/Phrase |
| S2 | cervical AND postoperative AND ehealth | Boolean/Phrase |
| S3 | cervical AND postoperative AND mhealth | Boolean/Phrase |
| S4 | cervical AND postoperative AND remote | Boolean/Phrase |
| S5 | cervical AND postoperative AND internet | Boolean/Phrase |
| S6 | cervical AND postoperative AND mobile | Boolean/Phrase |

171 references were included from ScienceDirect, PubMed and Web of Science

**Search strategy**

| **Search ID#** | **Search Terms** | **Search Options** |
| --- | --- | --- |
| S1 | thoracic AND surgery AND tele | Boolean/Phrase |
| S2 | thoracic AND surgery AND ehealth | Boolean/Phrase |
| S3 | thoracic AND surgery AND mhealth | Boolean/Phrase |
| S4 | thoracic AND surgery AND remote | Boolean/Phrase |
| S5 | thoracic AND surgery AND internet | Boolean/Phrase |
| S6 | thoracic AND surgery AND mobile | Boolean/Phrase |

566 references were included from ScienceDirect, PubMed and Web of Science

**Search strategy**

| **Search ID#** | **Search Terms** | **Search Options** |
| --- | --- | --- |
| S1 | thoracic AND operation AND tele | Boolean/Phrase |
| S2 | thoracic AND operation AND ehealth | Boolean/Phrase |
| S3 | thoracic AND operation AND mhealth | Boolean/Phrase |
| S4 | thoracic AND operation AND remote | Boolean/Phrase |
| S5 | thoracic AND operation AND internet | Boolean/Phrase |
| S6 | thoracic AND operation AND mobile | Boolean/Phrase |

115 references were included from ScienceDirect, PubMed and Web of Science

**Search strategy**

| **Search ID#** | **Search Terms** | **Search Options** |
| --- | --- | --- |
| S1 | thoracic AND discectomy AND tele | Boolean/Phrase |
| S2 | thoracic AND discectomy AND ehealth | Boolean/Phrase |
| S3 | thoracic AND discectomy AND mhealth | Boolean/Phrase |
| S4 | thoracic AND discectomy AND remote | Boolean/Phrase |
| S5 | thoracic AND discectomy AND internet | Boolean/Phrase |
| S6 | thoracic AND discectomy AND mobile | Boolean/Phrase |

3 references were included from ScienceDirect, PubMed and Web of Science

**Search strategy**

| **Search ID#** | **Search Terms** | **Search Options** |
| --- | --- | --- |
| S1 | thoracic AND decompression AND tele | Boolean/Phrase |
| S2 | thoracic AND decompression AND ehealth | Boolean/Phrase |
| S3 | thoracic AND decompression AND mhealth | Boolean/Phrase |
| S4 | thoracic AND decompression AND remote | Boolean/Phrase |
| S5 | thoracic AND decompression AND internet | Boolean/Phrase |
| S6 | thoracic AND decompression AND mobile | Boolean/Phrase |

28 references were included from ScienceDirect, PubMed and Web of Science

**Search strategy**

| **Search ID#** | **Search Terms** | **Search Options** |
| --- | --- | --- |
| S1 | thoracic AND postoperative AND tele | Boolean/Phrase |
| S2 | thoracic AND postoperative AND ehealth | Boolean/Phrase |
| S3 | thoracic AND postoperative AND mhealth | Boolean/Phrase |
| S4 | thoracic AND postoperative AND remote | Boolean/Phrase |
| S5 | thoracic AND postoperative AND internet | Boolean/Phrase |
| S6 | thoracic AND postoperative AND mobile | Boolean/Phrase |

230 references were included from ScienceDirect, PubMed and Web of Science

**Search strategy**

| **Search ID#** | **Search Terms** | **Search Options** |
| --- | --- | --- |
| S1 | lumbar AND surgery AND tele | Boolean/Phrase |
| S2 | lumbar AND surgery AND ehealth | Boolean/Phrase |
| S3 | lumbar AND surgery AND mhealth | Boolean/Phrase |
| S4 | lumbar AND surgery AND remote | Boolean/Phrase |
| S5 | lumbar AND surgery AND internet | Boolean/Phrase |
| S6 | lumbar AND surgery AND mobile | Boolean/Phrase |

426 references were included from ScienceDirect, PubMed and Web of Science

**Search strategy**

| **Search ID#** | **Search Terms** | **Search Options** |
| --- | --- | --- |
| S1 | lumbar AND operation AND tele | Boolean/Phrase |
| S2 | lumbar AND operation AND ehealth | Boolean/Phrase |
| S3 | lumbar AND operation AND mhealth | Boolean/Phrase |
| S4 | lumbar AND operation AND remote | Boolean/Phrase |
| S5 | lumbar AND operation AND internet | Boolean/Phrase |
| S6 | lumbar AND operation AND mobile | Boolean/Phrase |

52 references were included from ScienceDirect, PubMed and Web of Science

**Search strategy**

| **Search ID#** | **Search Terms** | **Search Options** |
| --- | --- | --- |
| S1 | lumbar AND discectomy AND tele | Boolean/Phrase |
| S2 | lumbar AND discectomy AND ehealth | Boolean/Phrase |
| S3 | lumbar AND discectomy AND mhealth | Boolean/Phrase |
| S4 | lumbar AND discectomy AND remote | Boolean/Phrase |
| S5 | lumbar AND discectomy AND internet | Boolean/Phrase |
| S6 | lumbar AND discectomy AND mobile | Boolean/Phrase |

53 references were included from ScienceDirect, PubMed and Web of Science

**Search strategy**

| **Search ID#** | **Search Terms** | **Search Options** |
| --- | --- | --- |
| S1 | lumbar AND decompression AND tele | Boolean/Phrase |
| S2 | lumbar AND decompression AND ehealth | Boolean/Phrase |
| S3 | lumbar AND decompression AND mhealth | Boolean/Phrase |
| S4 | lumbar AND decompression AND remote | Boolean/Phrase |
| S5 | lumbar AND decompression AND internet | Boolean/Phrase |
| S6 | lumbar AND decompression AND mobile | Boolean/Phrase |

83 references were included from ScienceDirect, PubMed and Web of Science

**Search strategy**

| **Search ID#** | **Search Terms** | **Search Options** |
| --- | --- | --- |
| S1 | lumbar AND postoperative AND tele | Boolean/Phrase |
| S2 | lumbar AND postoperative AND ehealth | Boolean/Phrase |
| S3 | lumbar AND postoperative AND mhealth | Boolean/Phrase |
| S4 | lumbar AND postoperative AND remote | Boolean/Phrase |
| S5 | lumbar AND postoperative AND internet | Boolean/Phrase |
| S6 | lumbar AND postoperative AND mobile | Boolean/Phrase |

169 references were included from ScienceDirect, PubMed and Web of Science

**Search strategy**

| **Search ID#** | **Search Terms** | **Search Options** |
| --- | --- | --- |
| S1 | spine AND surgery AND tele | Boolean/Phrase |
| S2 | spine AND surgery AND ehealth | Boolean/Phrase |
| S3 | spine AND surgery AND mhealth | Boolean/Phrase |
| S4 | spine AND surgery AND remote | Boolean/Phrase |
| S5 | spine AND surgery AND internet | Boolean/Phrase |
| S6 | spine AND surgery AND mobile | Boolean/Phrase |

786 references were included from ScienceDirect, PubMed and Web of Science

**Search strategy**

| **Search ID#** | **Search Terms** | **Search Options** |
| --- | --- | --- |
| S1 | spine AND operation AND tele | Boolean/Phrase |
| S2 | spine AND operation AND ehealth | Boolean/Phrase |
| S3 | spine AND operation AND mhealth | Boolean/Phrase |
| S4 | spine AND operation AND remote | Boolean/Phrase |
| S5 | spine AND operation AND internet | Boolean/Phrase |
| S6 | spine AND operation AND mobile | Boolean/Phrase |

93 references were included from ScienceDirect, PubMed and Web of Science

**Search strategy**

| **Search ID#** | **Search Terms** | **Search Options** |
| --- | --- | --- |
| S1 | spine AND discectomy AND tele | Boolean/Phrase |
| S2 | spine AND discectomy AND ehealth | Boolean/Phrase |
| S3 | spine AND discectomy AND mhealth | Boolean/Phrase |
| S4 | spine AND discectomy AND remote | Boolean/Phrase |
| S5 | spine AND discectomy AND internet | Boolean/Phrase |
| S6 | spine AND discectomy AND mobile | Boolean/Phrase |

54 references were included from ScienceDirect, PubMed and Web of Science

**Search strategy**

| **Search ID#** | **Search Terms** | **Search Options** |
| --- | --- | --- |
| S1 | spine AND decompression AND tele | Boolean/Phrase |
| S2 | spine AND decompression AND ehealth | Boolean/Phrase |
| S3 | spine AND decompression AND mhealth | Boolean/Phrase |
| S4 | spine AND decompression AND remote | Boolean/Phrase |
| S5 | spine AND decompression AND internet | Boolean/Phrase |
| S6 | spine AND decompression AND mobile | Boolean/Phrase |

120 references were included from ScienceDirect, PubMed and Web of Science

**Search strategy**

| **Search ID#** | **Search Terms** | **Search Options** |
| --- | --- | --- |
| S1 | spine AND postoperative AND tele | Boolean/Phrase |
| S2 | spine AND postoperative AND ehealth | Boolean/Phrase |
| S3 | spine AND postoperative AND mhealth | Boolean/Phrase |
| S4 | spine AND postoperative AND remote | Boolean/Phrase |
| S5 | spine AND postoperative AND internet | Boolean/Phrase |
| S6 | spine AND postoperative AND mobile | Boolean/Phrase |

243 references were included from ScienceDirect, PubMed and Web of Science

**Total result literature searches:	N = 3806 references.**
